# Tiny Earth: A Big Idea for STEM Education and Antibiotic Discovery

**DOI:** 10.1128/mBio.03432-20

**Published:** 2021-02-16

**Authors:** Amanda Hurley, Marc G. Chevrette, Deepa D. Acharya, Gabriel L. Lozano, Manuel Garavito, Jen Heinritz, Luis Balderrama, Mara Beebe, Martel L. DenHartog, Kamiyah Corinaldi, Renee Engels, Alyssa Gutierrez, Orli Jona, Josephine H. I. Putnam, Brody Rhodes, Tiffany Tsang, Simon Hernandez, Carol Bascom-Slack, Jessamina E. Blum, Paul A. Price, Debra Davis, Joanna Klein, Joshua Pultorak, Nora L. Sullivan, Nigel J. Mouncey, Pieter C. Dorrestein, Sarah Miller, Nichole A. Broderick, Jo Handelsman

**Affiliations:** a Wisconsin Institute for Discovery and Department of Plant Pathology, University of Wisconsin-Madison, Madison, Wisconsin, USA; b Division of Infectious Diseases and Division of Gastroenterology, Department of Pediatrics, Boston Children’s Hospital and Harvard Medical School, Boston, Massachusetts, USA; c Hamline University, Minneapolis, Minnesota, USA; d Wisconsin Institute for Discovery, University of Wisconsin-Madison, Madison, Wisconsin, USA; e Colorado State University, Fort Collins, Colorado, USA; f University of Alabama, Tuscaloosa, Alabama, USA; g University of West Alabama, Livingston, Alabama, USA; h Department of Molecular, Cellular, and Developmental Biology, Yale University, New Haven, Connecticut, USA; i University of Rochester Medical Center, Rochester, New York, USA; j Department of Medical Education, Tufts University, Boston, Massachusetts, USA; k University of Minnesota Medical School, Minneapolis, Minnesota, USA; l Department of Biology, Eastern Michigan University, Ypsilanti, Michigan, USA; m Department of Biology, Wingate University, Wingate, North Carolina, USA; n Department of Biology and Biochemistry, University of Northwestern-St. Paul, Saint Paul, Minnesota, USA; o Biology Department, Citrus College, Glendora, California, USA; p Joint Genome Institute, Berkeley, California, USA; q Collaborative Mass Spectrometry Innovation Center, Skaggs School of Pharmacy and Pharmaceutical Sciences, University of California San Diego, La Jolla, California, USA; r Department of Molecular and Cell Biology, University of Connecticut, Mansfield, Connecticut, USA; s Department of Biology, Johns Hopkins University, Baltimore, Maryland, USA; University of Connecticut

**Keywords:** antimicrobial activity, crowdsourcing, multiomics

## Abstract

The world faces two seemingly unrelated challenges—a shortfall in the STEM workforce and increasing antibiotic resistance among bacterial pathogens. We address these two challenges with Tiny Earth, an undergraduate research course that excites students about science and creates a pipeline for antibiotic discovery.

## OBSERVATION

Many calls to action have exhorted the scientific community to address the shortage of STEM college graduates that threatens the health of the scientific workforce (1, 2). Despite strong supporting evidence and the esteemed experts issuing these calls, they have not led to widespread adoption of teaching methods known to enhance retention of STEM undergraduates. It is time to match the policy imperative with an understanding of the impediments and strategies that address them, in order to enable consequential action in higher education.

At the same time that we are challenged by the STEM workforce dilemma, we also face a global shortage of antibiotics to treat the human pathogens that are accumulating alarming combinations of resistance genes. Although the STEM workforce and antibiotic resistance appear unrelated, we have designed a program that addresses both.

An insufficient number of STEM college graduates results, in part, from teaching methods that discourage many students from pursuing STEM majors (3). Introductory courses often fail to capture the nature of scientific inquiry, perpetuating a disconnect between discovery and knowledge. Students are instead led to believe that science is about mastering established facts rather than exploring the unknown. Traditional lecture formats favor majority students, disadvantaging women and ethnic minorities (4)—the very students needed by the STEM workforce and who comprise the majority of college students. Continued reliance on less effective teaching methods is astonishing given decades of compelling evidence that inquiry-based learning is a superior way for most students to learn (1, 3–7). Scientists should be swayed by evidence.

Research courses are a powerful tool for changing STEM in higher education. Such courses engage students in original research that captures the thrill of discovery and creates a sense of project ownership (7–9), both of which work to bolster students’ identification as scientists, an important aspect of remaining in STEM. In 2014, Freeman et al. (10) argued that continued use of classrooms in which students are taught by lecturing as a control treatment in research studies on active learning is inappropriate because the evidence in support of active learning is incontrovertible. Analogous to the ethical termination of a placebo control in a clinical trial in which the drug’s efficacy is unquestionable, it is time to implement research courses. The benefits of research courses—improving retention of students in STEM majors, learning, and gender and ethnic equity in the classroom (6, 11)—have produced national recommendations for broad integration of research courses into college education (1, 2, 12). Action responding to these appeals has been impeded by a litany of objections, including cost, the challenge of integrating an existing research course into a given curriculum, concerns about maintaining the quality of research experiences at nonresearch institutions, and the belief that undergraduate students cannot make meaningful contributions to research or lack sufficient knowledge to engage in it at all. Consequently, most introductory college science education continues to omit research experiences and is didactic rather than interactive or student-centered (13). We provide an example research course to illustrate to leaders in higher education that the objections can be addressed, and it is time to unite around the bold goal of offering every college student the opportunity to take a research course.

To demonstrate the feasibility of widespread implementation of research courses, we developed Tiny Earth, a course focused on antibiotic discovery. It is based on other successful research courses (7, 9, 14, 15) and addresses common barriers to large-scale implementation. In particular, we modeled Tiny Earth on the structure of the pioneering SEA PHAGES program (7), a highly successful course on bacteriophage discovery with a program that trains instructors. We also used several of our own well-established programs as models for instructor training (5, 16). To make the course accessible to diverse institutions and instructors, we addressed the issues of cost, feasibility, and fidelity. Tiny Earth is taught to more than 10,000 students per year in 27 countries, which speaks to the adaptability of the course and success of our implementation strategy. Here, we outline that strategy and demonstrate the meaningful contributions of undergraduates to a powerful pipeline for antibiotic discovery.

**FIG 1:**
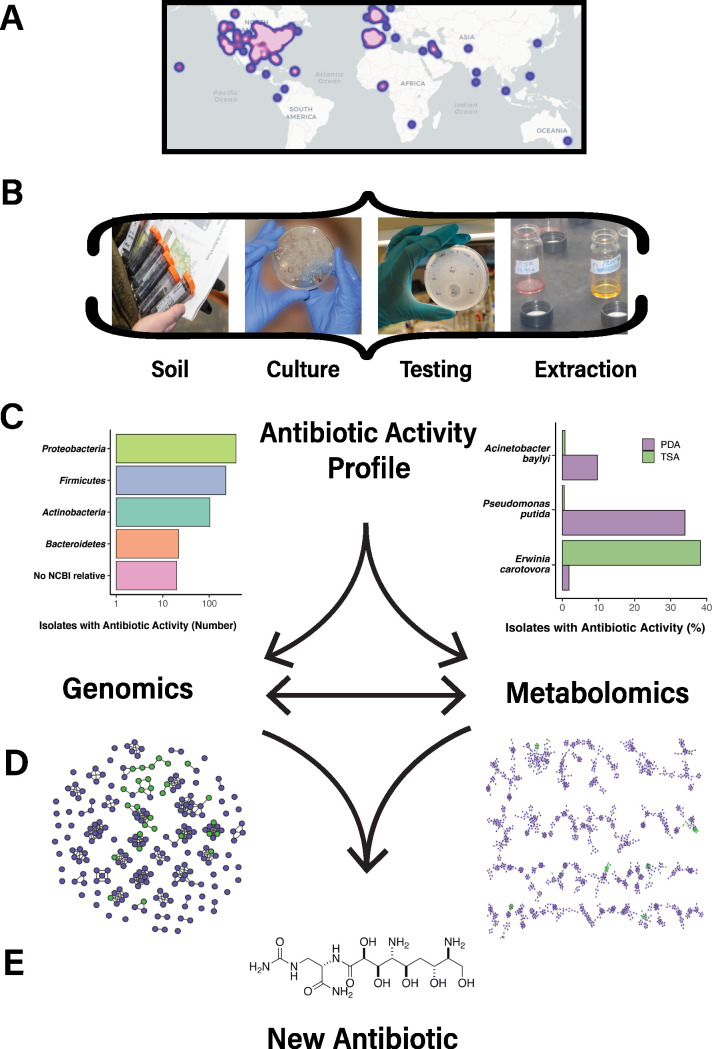
Tiny Earth Chemistry Hub pipeline. (A) Heat map of global Tiny Earth partner institutions. (B) Strains are catalogued and stored at the Chemistry Hub where each strain is then tested against the safe relatives of ESKAPE pathogens to verify activity profile. Molecules from active strains are extracted in organic solvents. (C, left) Phylum affiliations of strains submitted to the Chemistry Hub as determined by 16S rRNA gene sequencing. NCBI (National Center for Biotechnology Information) catalogues all known sequenced organisms. (C, right) Growth medium differentially affects discovery rate of antibiotic-producing bacteria based on target bacteria. Tryptic soy agar (TSA) enhances isolation of bacteria that inhibit Erwinia carotovora, while potato dextrose agar (PDA) favors inhibition of Acinetobacter baylyi and Pseudomonas putida. Results shown for the three target bacteria are from three separate soils, suggesting soil origin influences media specificity. (D, left) DNA is extracted for whole-genome sequencing and chemical compounds are harvested for mass determination to search for novel patterns. Putative biosynthetic pathways for potential antibiotics are organized in clusters by similarity, with green circles representing similarity to pathways of known function and purple indicating those of unknown function. (D, right) Families of molecules produced by sequenced strains with the same color legend as the left panel. (E) Using both genomic and metabolomic data, strains with potentially novel chemistry are prioritized to purify molecules and determine the structure and activity of a new antibiotic; the structure of soil-derived zwittermicin (37) is shown as an example.

**Course overview.** We are in a postantibiotic era (17) in which bacteria are becoming increasingly resistant to our current arsenal of antibiotics. Antimicrobial-resistant infections are predicted to kill 10 million people per year by 2050 (18). The COVID-19 pandemic may exacerbate the global burden of antimicrobial resistance, as 72% of patients hospitalized with the viral disease are prescribed antibiotics, when only 7% have bacterial coinfections (19). To inspire students with the power of science to address real-world problems, Tiny Earth provides students with a global community that shares the goal of fighting infectious disease (Fig. 1A). Tiny Earth is a platform for teaching biology content through research in which students generate and test hypotheses about antibiotic-producing bacteria in soil (the source of 75% of antibiotics in current use) (20). They culture bacteria from soil, screen them for antibiotic activity, and characterize their biochemical profiles and 16S rRNA gene sequences to determine taxonomic assignment (Fig. 1B). The students extract metabolites from the cultures with organic solvents and test the extracts for antibiotic activity. Since time constraints and access to equipment often preclude further characterization by the students, the University of Wisconsin-Madison established the Tiny Earth Chemistry Hub in 2018, which characterizes the genomes and metabolomes of the strains submitted by instructors and then prioritizes them for further chemical analysis. The Tiny Earth Chemistry Hub is funded through a combination of philanthropy, state, and university funding, and a small startup to capitalize on the fruits of the network labor, but is currently seeking more sustainable support. Strains are tracked in the Tiny Earth Database (https://data.tinyearth.wisc.edu/), which is available to all students and instructors in the network. The course can be taught at lower cost than typical introductory biology laboratories, making it accessible to institutions with severely limited budgets.

**Adaptability to diverse institutions.** Objections to research courses include concern about their adaptability to nonresearch universities, particularly instructor expertise and facilities. We address instructor expertise by providing a week-long training program at no cost other than travel, at the University of Connecticut and at the University of Wisconsin-Madison. The cost of training is low—between $500 to $1,300 per instructor—and has been covered by gifts from the biotechnology industry, whose own workforce challenges generate an appreciation for the goal of producing more STEM college graduates. The training, led by experts in both microbiology and science education, provides pedagogical foundations, instructional materials, and hands-on laboratory practice, enabling instructors from various fields to teach the course. Instructor training focuses on inclusive teaching strategies to ensure that diverse students have access to the research experience.

Each newly trained instructor is strategically paired with a more experienced Tiny Earth Partner Instructor (TEPI) mentor who provides advice and troubleshooting during course implementation. TEPIs are kept abreast of new instructional developments through workshops, events, newsletters, web content, and social media. Tiny Earth has been implemented by more than 700 instructors at tribal colleges, community colleges, universities, liberal arts colleges, and technical colleges, indicating its versatility and adaptability, and interactions among instructors from diverse settings enhances the dynamic, creative network.

**Facilities and safety.** Tiny Earth addresses the concern about whether diverse institutions have appropriate facilities to conduct research or to do it safely by requiring simple and inexpensive equipment and tailoring experimental protocols so that they pose little risk. For instance, student safety in the absence of biosafety cabinets is addressed by techniques to minimize microbial risk. The bacteria used in screening for antibiotic activity are safe (risk group level 1) relatives of the ESKAPE pathogens, the six bacterial species thought to pose especially high risk to human health because of their escalating antibiotic resistance (21). To address concern about culturing human pathogens from soil, methods align with the American Society for Microbiology’s biosafety guidelines (22). All protocols involve growing isolates on solid medium, which is less likely than liquid culture to produce the bacterial aerosols that provide routes for infection by several human pathogens. Instructor training includes biosafety, risk mitigation strategies, and best practices for working with unknown bacterial isolates, ensuring that even nonmicrobiologists can lead the course safely.

**Student contributions to research.** Some scientists object to the notion that undergraduates can make meaningful research contributions in one semester, and argue that without significant results, the experience is no better than a typical laboratory course. Moreover, many have suggested that first- or second-year students lack sufficient knowledge to participate meaningfully in research. The evidence contradicts these arguments. Even if they do not discover the next penicillin, Tiny Earth students contribute significant scientific knowledge. A key finding, for example, is the extraordinary metabolic diversity still to be discovered in soil, thereby challenging a widely held dogma in the field. Since the 1980s, most large pharmaceutical companies have closed their antibiotic discovery units, often arguing that the soil is “tapped out,” leaving few if any antibiotics undiscovered (23). Scant publicly available data support the claim, and several recent discoveries demonstrate that new methodology provides access to unprecedented molecular diversity (24). Inspired by the potential of 10^4^ to 10^6^ species per gram of soil (25, 26), Tiny Earth students use modern knowledge and their own innovation to prove the dogma wrong.

Tiny Earth students tap deeply into unknown diversity by collecting soil in previously unexplored locations, focusing on bacterial phyla that were traditionally sidelined by industry (Fig. 1C, left panel), and employing creative and alternative methods. Students discovered that potato dextrose agar medium (to our knowledge not used in past mainstream antibiotic discovery) increases the frequency of antibiotic producers compared to more common culture conditions (Fig. 1C, right panel). Recent research shows that bacterial coculture triggers antibiotic production in many organisms (27), a finding that students have exploited for discovery. Tiny Earth students also seek antibiotics that inhibit nontraditional target organisms, such as Acinetobacter, which emerged as health threats after industry largely shuttered antibiotic-discovery laboratories.

Industry has often argued that the antibiotic-discovery pipeline is too expensive, time-consuming, and uncertain. Part of the concern is the laborious process traditionally required to determine whether the molecule responsible for activity is new or already known. That work is perfectly suited for the undergraduate workforce, which is renewed annually before burnout can set in, and shares the cost of discovery with the cost of education. Students enter their data into a shared database available to all members of the network. Data gathered subsequently by the Tiny Earth Chemistry Hub are also made available in the database. The Hub uses metabolomic (Fig. 1D, right panel) characterization of molecular families in crude extracts to identify known molecules, obviating the need to purify every previously discovered active molecule (28, 29). Genomic sequencing (Fig. 1D, left panel) further sharpens the focus by identifying isolates that contain genes encoding previously unknown biosynthetic pathways (30). Triangulating biological activity, metabolomic profiles, and genomic analysis, Tiny Earth prioritizes candidate compounds before purification and structure determination (Fig. 1), thereby streamlining discovery. Public access to all Tiny Earth data enables other researchers to mine it for new insights (https://data.tinyearth.wisc.edu/public_database). Each discovery is made possible by the research contributions of undergraduates, dispelling the notion that undergraduates cannot contribute meaningfully to research in one semester early in their college education.

**Discussion.** It is time for disruptive innovation in both college STEM education and antibiotic discovery. Calls for change in teaching methods have persisted for more than 20 years (12, 31–33), during which evidence indicating the superior outcomes of active and inquiry-based learning has become irrefutable (2, 10). Tiny Earth and its predecessors, such as SEA PHAGES, demonstrate the ease of broad implementation of research courses in every type of institution of higher learning. The University of Texas-Austin (UT-Austin) illustrated the feasibility of teaching research courses to large student populations by implementing a research course requirement for their entire freshman class of almost 9,000 students (34). UT–Austin’s freshman research course improved learning and college success, and increased students’ future earning potential (35). If a massive public university can accomplish the remarkable feat of offering research experience to all first-year students, many other institutions will be able to as well.

Likewise, calls for stepping up the pace of antibiotic discovery are issued regularly (18), and yet large pharmaceutical companies have largely abandoned the effort and small biotech companies struggle and frequently expire. The Tiny Earth students may access previously unknown chemical diversity and isolation methods, reducing the initial cost of discovery and producing uncommon candidate antibiotics.

The National Science Foundation and the Howard Hughes Medical Institute have spoken on the importance of research courses by investing heavily in their development, evaluation, and dissemination. The National Academies and the White House advocated implementation of undergraduate research courses to enhance STEM education and the workforce (2, 36). It is time for institutions of higher education to step up, heed the compelling research, and make research courses standard in the undergraduate curriculum. The resources exist. Budgets for traditional introductory biology laboratory exercises should be redirected to original research courses. Consortia of college and university leadership should collaborate to identify and address common barriers and affect curricular change collectively. These consortia could be convened by regional groups such as the Big 10 Academic Alliance or organizations of similar institutions, such as the Association of American Universities, Association of Public and Land-grant Universities, and the American Association of Community Colleges. Higher education has a responsibility to provide the United States and the rest of the world with a sufficient STEM workforce, college students deserve meaningful STEM experiences, and we will all benefit from the research results they produce. With feasible, vetted, effective, and inclusive research experiences like Tiny Earth, the question is, what are we waiting for?
